# Prevalence, pattern and prognosis of lung lesions in pediatric Hodgkin lymphoma

**DOI:** 10.1038/s41598-026-60214-5

**Published:** 2026-07-06

**Authors:** Jonas Steglich, Andishe Attarbaschi, Auke Beishuizen, Michaela Cepelova, Karin Dieckmann, Ana Fernández-Teijeiro, Jamie E. Flerlage, Alexander Fosså, Dirk Hasenclever, Andrea Hraskova, Tomasz Klekawka, Dieter Körholz, Lars Kurch, Judith Landman-Parker, Thierry Leblanc, Christiane Ludwig, Christine Mauz-Körholz, Anne Uyttebroeck, Dirk Vordermark, William Hamish Wallace, Walter A. Wohlgemuth, Dietrich Stoevesandt

**Affiliations:** 1 Department of Radiology, University Hospital Halle, Ernst-Grube-Straße 40, 06120 Halle/Saale, Germany; 2 Department of Pediatric Hematology and Oncology, St. Anna Children’s Hospital, Medical University of Vienna, Vienna, Austria; 3 St. Anna Children’s Cancer Research Institute, Vienna, Austria; 4 Princess Máxima Center for Pediatric Oncology, Utrecht, The Netherlands; 5 Department of Pediatric Hematology and Oncology, University Hospital Motol, Prague, Czech Republic; 6 Medical Faculty of Charles University, Prague, Czech Republic; 7 Department of Radiation Oncology, University Hospital Vienna, Vienna, Austria; 8 Pediatric Onco-Hematology Unit, Sociedad Española de Hematología y Oncología Pediátricas (SEHOP), Hospital Universitario Virgen Macarena, Sevilla University, Seville, Spain; 9 Department of Pediatrics, University of Rochester Medical Center, Rochester, NY USA; 10 Department of Medical Oncology and Radiotherapy, Oslo University Hospital, Oslo, Norway; 11 Institute of Medical Informatics, Statistics and Epidemiology, University of Leipzig, Leipzig, Germany; 12 Department of Pediatric Hematology and Oncology, University Children’s Hospital, Bratislava, Slovakia; 13 Department of Pediatric Oncology and Hematology, University Children’s Hospital of Kraków, Kraków, Poland; 14 Department of Pediatric Hematology and Oncology, University Hospital Giessen-Marburg, Giessen, Germany; 15 Department of Nuclear Medicine, University of Leipzig, Leipzig, Germany; 16 Hôpital Armand- Trousseau Sorbonne Université, Paris, France; 17 Service d’Hématologie Pédiatrique, Hôpital Robert-Debré and Université Paris-Cité, Paris, France; 18 Department of Internal Medicine, University Hospital Halle, Halle/Saale, Germany; 19 Medical Faculty of the Martin-Luther-University of Halle-Wittenberg, Halle/Saale, Germany; 20 Department of Pediatric Hematology and Oncology, University Hospitals Leuven, KU Leuven, Leuven, Belgium; 21 Department of Radiation Oncology, Medical Faculty of the Martin-Luther- University, Halle/Saale, Germany; 22 Department of Paediatric Oncology, Royal Hospital for Sick Children, University of Edinburgh, Edinburgh, UK

**Keywords:** Hodgkin lymphoma, Lung involvement, Lung staging, Computed tomography, Pediatric oncology, Cancer imaging, Hodgkin lymphoma, Paediatric research, Computed tomography

## Abstract

Children and adolescents with classical Hodgkin lymphoma have a progression-free survival (PFS) rate of ≥ 90% with current treatments. The current challenge is to reduce adverse late-effects while maintaining high survival. Distinguishing lung involvement (Stage IV) from benign lung lesions remains a staging challenge in pediatric Hodgkin lymphoma (pHL). This study analyzed the prevalence, morphological patterns, and prognostic impact of lung lesions on progression-free survival (PFS) within the EuroNet-PHL-C1 trial. A retrospective analysis was conducted on chest CT scans from 1,298 pHL patients enrolled in the EuroNet-PHL-C1 trial. Patients were stratified by established treatment groups (TG-1, TG-2, TG-3). Lesions were classified by morphological pattern, and Kaplan-Meier analysis was used to compare 60-month PFS between groups with and without lung lesions. Lung lesions were identified in 60.2% (782/1298) of patients, with nodules being the predominant pattern (89%). In the combined TG-1 and TG-2 cohort (early/intermediate stages), the presence of any lung lesion correlated with significantly lower 5-year PFS (85.5% vs. 91.7%; *p* = 0.0197). Importantly, this lower PFS was driven by non-nodule morphologies. In the TG-3 (advanced stage) cohort, neither the presence of lung lesions nor stage IV classification significantly affected PFS. Lung lesions are highly prevalent in pHL. However, the presence of pulmonary nodules does not confer an inferior prognosis. The prognostic impact of lung lesions is primarily limited to non-nodule patterns in early-stage disease. These findings suggest that incorporating morphological patterns may be beneficial for refining risk stratification in future pHL trials.

## Introduction

Pediatric Hodgkin lymphoma (pHL) has become one of the most curable cancers with survival rates exceeding 90% ^1–8^. Therefore, the current challenge in this age group is to reduce adverse therapeutic late effects while maintaining high cure rates^9–13^. The European Network for Pediatric HL (EuroNet-PHL) conducted the EuroNet-PHL-C1 trial focused on preventing both overtreatment and undertreatment by risk and response adapted therapy^7,8^. For this trial, patients were assigned to one of three treatment groups (TG) based on the extent of disease and presence or absence of B-symptoms.

Staging is based on the Ann Arbor classification^14^ with Cotswold and Lugano modifications^15–17^. Current staging recommendations combine computed tomography (CT) and/or magnetic resonance imaging (MRI) with ^18^Fluorodeoxyglucose positron emission tomography (^18^FDG-PET)^18^ to determine nodal sites and extranodal organs affected.

The lung is considered one of the most affected extranodal sites in pHL. Frequency of lung involvement of classical HL (cHL) ranges from 9% to 43%, depending on the imaging method, age group^19–24^ and staging definition^25,26^ and is easier to identify with modern imaging. Given the high prevalence of incidental lung lesions in healthy children^27^, careful differentiation between nonspecific pulmonary findings and pHL-related lung involvement consistent with Ann Arbor stage IV disease is essential, as stage IV classification stratifies patients into higher-risk treatment cohorts associated with potentially increased adverse therapeutic late effects. Consequently, a distinction needs to be made between stage-IV-qualifying and non-stage-IV-qualifying foci, which means that not every lung lesion is consistent with lung involvement. The EuroNet-PHL-C1 trial, similar to other trials, defined lung involvement for staging purposes based solely on morphologic criteria from CT, specifically the size and number of lung lesions, rather than metabolic activity^28^. This approach is rooted in the recognition that CT offers superior sensitivity for detecting small lung lesions compared to PET, especially given the limitations of early PET/CT technology^29–33^. According to the EuroNet-PHL-C1 staging definitions, lung involvement was identified if a patient presents with > 3 intrapulmonary lung lesions, each with a diameter less than 10 mm, or at least one intrapulmonary lesion with a diameter of 10 mm or greater. This approach was based on a consensus among the trial authors, which was intended to avoid upstaging due to incidental lung lesions, but was arbitrary and not based on survival data.

It is important to distinguish stage-IV-defining lung lesions from E-lesions, which represent contiguous infiltration of a lymph node mass into the lung parenchyma. ^34^ E-lesions are a distinct entity and considered a separate prognostic factor for risk assignment that can be associated with any stage of the Ann Arbor classification.

## Objectives

Optimization of the criteria for lung involvement in pediatric cHL requires a systematic analysis of the impact of lung lesions on survival. Based on data from the large multinational EuroNet-PHL-C1 trial, the aims of this analysis were:

1. To comprehensively describe the frequency and morphologic patterns of intrapulmonary lung lesions in pHL, and
2. To evaluate the prognostic value of lung lesions with regards to treatment intensity.

## Methods

### I. Description of study participants and data sources

In this retrospective analysis, we re-evaluated patients who participated in the EuroNet-PHL-C1 trial (NCT00433459; EudraCT 2006-000995-33) that recruited patients at 186 hospital sites across 16 European countries between 2009 and 2013. ^7,8^

Children and adolescents with newly diagnosed stage IA, IB, and IIA cHL < 18 years of age were designated early-stage and assigned to treatment group 1 (TG-1). Stages IAE and IBE, IIAE, IIB, or IIIA were classified as intermediate-stage and assigned to treatment group 2 (TG-2). Stages IIBE, IIIAE, IIIB, IIIBE, and all stages IV (A, B, AE, and BE) were designated advanced-stage and assigned to treatment group 3 (TG-3).

All patients were treated with two cycles of OEPA. Patients in TG-2 received two additional cycles and patients in TG-3 four additional cycles of consolidation chemotherapy, respectively. Patients in TG-2 and TG-3 were randomized to receive either COPP or COPDAC. COPDAC was identical to COPP except that dacarbazine replaced procarbazine. Radiotherapy (RT) was omitted in patients who responded adequately to OEPA. Adequate response was defined as partial or complete morphologic remission and complete metabolic remission according to IHP criteria (comparable to Deauville 1–2) ^33^. All patients with inadequate response received RT at a dose of 19.8 Gy to all initially involved tumor sites. ^7,8^

All patients and/or their legal guardians gave written informed consent. The institutional review board of the EuroNet-PHL-C1 trial approved this retrospective imaging data analysis and waived the requirement for additional informed consent. The study was performed in accordance with good clinical practice and the Declaration of Helsinki.

Out of 2102 EuroNet-PHL-C1 trial participants, staging images of 1752 patients (83.3%) underwent central review in Halle (Saale), Germany. Patients with incomplete scans or inadequate imaging quality were excluded (*n* = 454) from this analysis. Inadequate imaging quality was defined as significant respiratory or motion artifacts, incompletely captured lung parenchyma, slice thickness equal or larger than 10 mm, or low spatial resolution on ultra-low-dose CTs or low-dose CTs acquired only for attenuation correction of PET.

This analysis includes chest CT scans with adequate quality for 1298 patients. Images were reanalyzed for intrapulmonary lung lesions irrespective of the decisions of the central review board during the conduct of the trial about lung involvement. E-lesions resulting from contiguous infiltration of an adjacent lymph node mass into the lung, classified as E-lesions^34^, were not included as these are considered a distinct entity with different prognostic implications and staging considerations. The presence and pattern of lung lesions were analyzed by three physicians (D.S., radiologist, 15 years of experience; J.S., resident in radiology, 2 years of experience; C.L., specialist in internal medicine and pneumology; 7 years of experience). The image interpretation was performed in consensus, meaning each lesion was reviewed by all three readers, and initial disagreements were discussed and resolved to reach a unified interpretation before data entry. A comparison of the analyzed patients (*n* = 1298) with the non-analyzed patients (*n* = 804) from the EuroNet-PHL-C1 trial was made to check for selection bias using chi square test in gender, age, treatment group, erythrocyte sedimentation rate (ESR) ≥ 30 mm within the first hour, Ann Arbor stage, presence of B-symptoms, E-lesions and bulky disease, defined as a contiguous tumor volume of at least 200 ml.

### II. Procedures for data collection and analysis

For each CT examination, the reconstruction parameters slice thickness, increment, tube current and field of view were recorded. Each scan was assessed for the presence of any intrapulmonary lung lesion at standard lung window settings (center: -600 HU; width: 1500 HU), without regard to metabolic activity. All intrapulmonary lung lesions were documented regardless of whether they fulfilled the staging criteria for lung involvement.

Documentation of lung lesions was restricted to 10 per patient to limit the impact of individual patients with diffuse lung lesions on the pattern-based analysis. If different patterns were present, they were included in a ratio that reflected their frequency in the patient. Each discrete intrapulmonary lesion was systematically classified according to the currently recommended terms of the Fleischner Society^35^:

1. Nodules/masses, defined as circumscribed, typically round opacity, less than or equal to 30 mm in average diameter, while a rounded lesion of similar shape that is larger than 30 mm is called a mass^35^;
2. Consolidations, defined as opacity causing complete obscuration of the underlying bronchi and vessels;
3. Ground-glass opacities, defined as area of increased attenuation that does not completely obscure the underlying bronchial and vascular structures;
4. Parenchymal bands, defined as thin linear opacity paralleling the pleura, occasionally described as extending to the pleural surface, and.
5. Reticular opacities, defined as intersecting linear opacities that produce an appearance resembling a net.

The diameter of each lesion was measured in millimeters (mm) in its largest axial dimension. The bronchopulmonary segment in which the center of each lesion was located was determined. The relation of a lesion to the pleura or hilum on axial slices was defined using four groups: contact to the pleura, subpleural (distance to the pleura < 1 cm), centrilobular (distance to the pleura ≥ 1 cm) and close to the hilum.

### III. Endpoints

The primary endpoint was progression-free survival (PFS), defined as time from the start of treatment to the occurrence of the following events: death from any cause, progression or relapse of cHL, with a target rate of 90% at 5 years according to the EuroNet-PHL-C1 trial^8,36^. Estimates of 5-year rates with two-sided 95% confidence intervals (CIs) were obtained with the Kaplan Meier product limit estimator. The outcome was considered to be consistent with the target rate if the CI included or was higher than 90%.

### IV. Analysis strategy

As prognostic factors gradually lose their predictive value when treatment is successfully adapted to the disease burden^37^, separate analyses were performed for TG-1, TG-2 and TG-3 to account for treatment intensity in the PFS analysis.

For the combined analysis of TG-1 and TG-2, patients without lung lesions were compared with those who had lung lesions but did not meet the EuroNet-PHL-C1 staging criteria for lung involvement. An additional analysis for TG-1 and TG-2 compared patients presenting with pulmonary nodules to those exhibiting other patterns of lung lesions, neither of which was interpreted as lung involvement.

For the TG-3 analysis, patients classified as non–stage IV (IIBE, IIIAE, IIIB, or IIIBE) were compared with those classified as stage IV, with both cohorts further stratified by the presence or absence of lung lesions.

Within the stage IV cohort, comparisons were performed across the following subgroups:

- Stage IV patients without lung lesions but with involvement of at least one other organ;
- Stage IV patients with lung lesions meeting criteria for lung involvement and no additional organ involvement;
- Stage IV patients with lung lesions meeting criteria for lung involvement and involvement of at least one additional organ; and.
- Stage IV patients with lung lesions not meeting criteria for lung involvement but with involvement of at least one other organ.

The statistical analysis was conducted using R (The R Foundation for Statistical Computing, Version 4.0.2, Vienna, Austria).

## Results

### I. Summary of the analyzed cohort

**Fig. 1:**
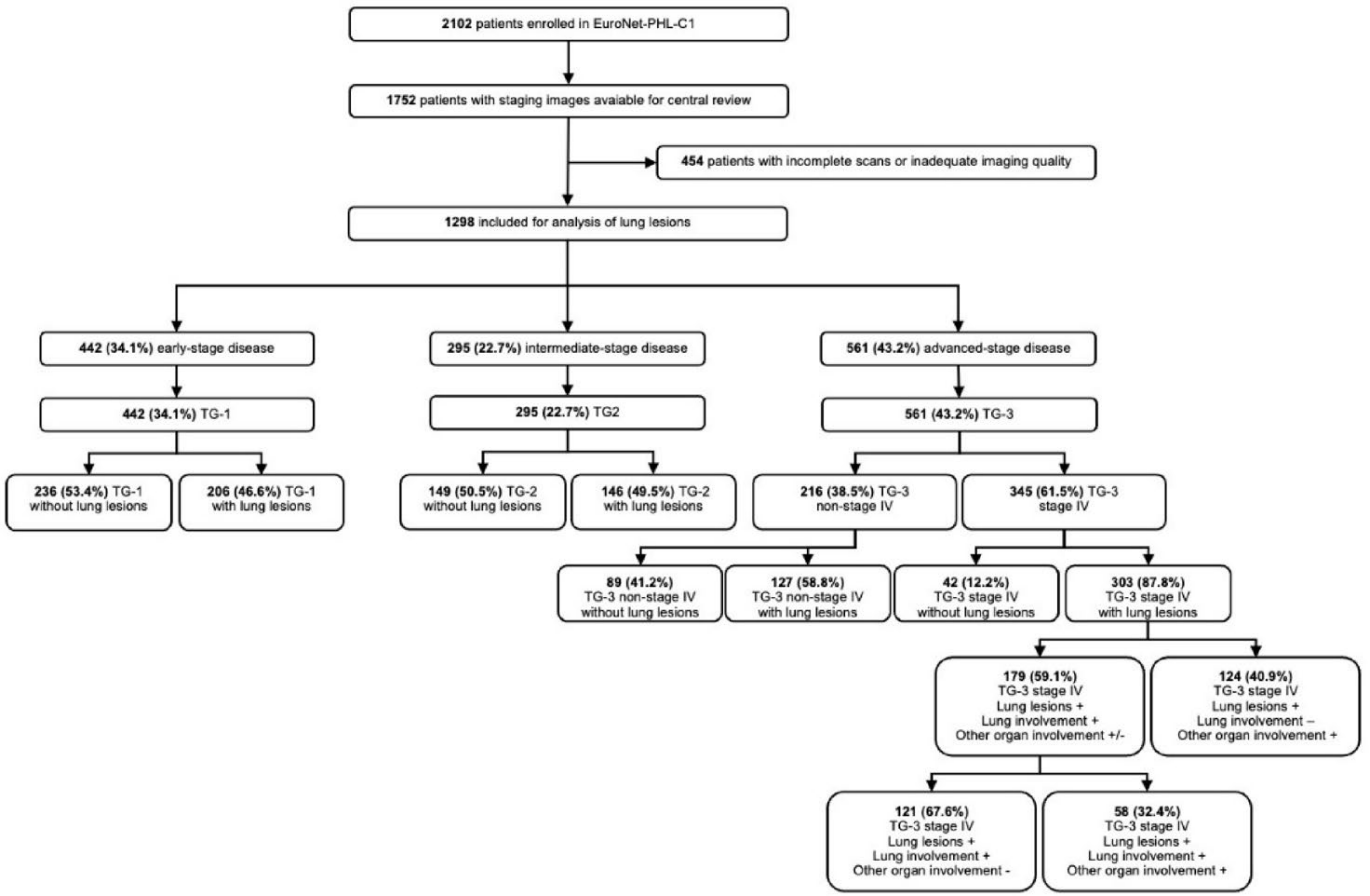
Process for selecting patients from the EuroNet-PHL-C1 trial for the analysis of progression-free survival. Of 2102 patients enrolled in the EuroNet-PHL-C1 trial, 1752 had images available for central review. As 454 patients had incomplete imaging data or insufficient scans, 1298 patients could be included in the analysis of the impact of lung lesions on progression-free survival, including 442 patients with early-stage disease in Treatment Group 1 (TG-1), 295 patients with intermediate-stage disease in Treatment Group 2 (TG-2), and 561 patients with advanced-stage disease in Treatment Group 3 (TG-3)

The analysis included 1298 patients with sufficient lung imaging available for central review (62%) out of 2102 patients in the EuroNet-PHL-C1 trial (Fig. 1).

An analysis for selection bias found no significant differences in gender (*p* = 0.343), age (*p* = 0.337), TG (*p* = 0.993), ESR (*p* = 0.449), Ann Arbor stage (*p* = 0.213), presence of B-symptoms (*p* = 0.603) or presence of E-lesions (*p* = 0.246) between the patients who were selected for this analysis and the group, that was excluded. The patients selected for this analysis presented with significantly (*p* < 0.001) more bulky disease (40.6%) than those not included (32.3%) (Table 1).

**Table 1:** Comparison of baseline characteristics of patients from the overall EuroNet-PHL-C1 trial and the proportion included for this analysis sorted by “with lung lesions” and “without lung lesions”

	Patients in the EuroNet-PHL-C1 titration study (*n* = 2102)	Patients excluded from the analysis (*n* = 804)	Patients included in the analysis (*n* = 1298)	Patients with lung lesions (*n* = 782)	Patients without lung lesions (*n* = 516)
Sex					
Male	1093 (52%)	407 (50.6%)	686 (52.9%)	431 (55.1%)	255 (49.9%)
Female	1009 (48%)	397 (49.4%)	612 (47.1%)	351 (44.9%)	261 (50.6%)
Age, years					
≥ 13 years	1481 (70.5%)	557 (69.3%)	924 (71.2%)	574 (73.4%)	350 (67.8%)
< 13 years	621 (29.5%)	247 (30.7%)	374 (28.8%)	208 (26.6%)	166 (32.2%)
Treatment group					
TG-1	714 (34%)	272 (33.8%)	442 (34.1%)	206 (26.3%)	236 (45.7%)
TG-2	479 (22.8%)	184 (22.9%)	295 (22.7%)	146 (18.7%)	149 (28.9%)
TG-3	909 (43.2%)	348 (43.3%)	561 (43.2%)	430 (55%)	131 (25.4%)
Erythrocyte sedimentation rate in the first hour ^1^ not valid for 629 patients; ^2^ not valid for 154 patients; ^3^ not valid for 475 patients; ^4^ not valid for 301 patients; ^5^ not valid for 174 patients					
< 30 mm	497 (33.7%)^1^	212 (32.6%)^2^	285 (34.6%)^3^	132 (27.4%)^4^	153 (44.7%)^5^
≥ 30 mm	976 (66.3%)^1^	438 (67.4%)^2^	538 (65.4%)^3^	349 (72.6%)^4^	189 (55.3%)^5^
Ann Arbor stage					
I	48 (2.3%)	21 (2.6%)	27 (2.1%)	16 (2%)	11 (2.1%)
II	1116 (53.1%)	435 (54.1%)	681 (52.5%)	322 (41.2%)	359 (69.6%)
III	407 (19.4%)	164 (20.4%)	245 (18.9%)	141 (18%)	104 (20.2%)
IV	531 (25.3%)	184 (22.9%)	345 (26.6%)	303 (38.7%)	42 (8.1%)
Presence of B-symptoms					
A: No	1258 (59.8%)	475 (59.1%)	783 (60.3%)	435 (55.6%)	348 (67.2%)
B: Yes	844 (40.2%)	329 (40.9%)	515 (39.7)	347 (44.4%)	168 (32.6%)
E-lesion					
No	1644 (78.2%)	640 (79.6%)	1004 (77.3%)	573 (73.3%)	431 (83.5%)
Yes	458 (21.8%)	164 (20.4)	294 (22.7%)	209 (26.7%)	85 (16.5%)
Bulky disease ^1^ not valid for 88 patients; ^2^ not valid for 77 patients; ^3^ not valid for 11 patients; ^4^ not valid for 7 patients; ^5^ not valid for 4 patients					
No	1257 (62.4%)^1^	492 (67.7%)	765 (59.4%)^3^	420 (54.2%)^4^	345 (67.4%)^5^
Yes	757 (37.6%)^1^	235 (32.3%)	522 (40.6%)^3^	355 (45.8%)^4^	167 (32.6%)^5^

Based on risk stratification, 442 (34.1%) patients analyzed were in TG-1, 295 (22.7%) in TG-2, and 561 (43.2%) in TG-3. Mean slice thickness was 3.5 mm, mean increment was 3.0 mm, mean tube voltage was 120 kV and mean field of view was 372 mm.

### II. Prevalence and morphologic pattern of lung lesions

Of the 1298 patients analyzed, 782 (60.2%) had at least one intrapulmonary lung lesion. Consequently, in 516 (39.8%) patients, no lung lesions were detected at baseline. The presence of lung lesions correlated significantly with high ESR (*p* = 0.004), Ann Arbor stage of disease (*p* < 0.001), the presence of B-symptoms (*p* = 0.018), the presence of E-lesions (*p* = 0.015) and the absence of bulky disease (*p* = 0.008) at baseline.

The number of lung lesions varied: At least one lung lesion was present at baseline in 206 of 442 patients in TG-1 (46.6% [41.9%; 51.4%]), 146 of 295 patients in TG-2 (49.5% [43.7%; 55.3%]), and 430 of 561 patients in TG-3 (76.7% [72.9%; 80.0%]) (Fig. 1). Overall, patients had a mean of 2.5 lung lesions (SD = 3.3). A total of 225 patients (17%) presented with a single lesion, 136 (10%) with two lesions, 85 (7%) with three lesions, 62 (5%) with four lesions, 34 (3%) with five lesions, 37 (3%) with six lesions, 24 (2%) with seven lung lesions, 20 (2%) with eight lesions, 15 (1%) with nine lung lesions, and 144 (11%) with ten or more lung lesions.

In some cases, a combination of different morphologic pattern was present: Out of 1298 patients, 729 (56.2%) presented with nodules, 76 (5.9%) presented with consolidations, 64 (4.9%) presented with ground-glass opacities, 89 (6.9%) presented with parenchymal bands and 21 (1.6%) patients presented with reticular opacities (Table 2). Although mixed morphologic patterns were observed in a minority of patients across all stages and treatment groups, nodules were the predominant finding throughout. Nodules were the sole morphologic pattern in 73.2% (573/782) of patients with lung lesions, whereas a combination of nodules with at least one additional pattern was present in 28.0% (156/557) of patients with two or more lesions.

**Table 2:** The number of patients and the frequency of lung lesions were analyzed according to their morphological pattern, stratified by Ann Arbor stage and therapy group. Percentages in parentheses refer to the proportion of patients within the respective Ann Arbor stage or treatment group. For example, among 347 patients with Ann Arbor stage IV disease, 38 exhibited at least one consolidation, corresponding to 11.0% of patients in this stage. Notably, individual patients could present with more than one morphological pattern; consequently, the sum of patients across lesion types may exceed the total number of patients within a given stage or treatment group

	Patients included in the analysis	Nodules/Masses	Consolidations	Ground-glass opacities	Parenchymal bands	Reticular opacities
Ann Arbor stage						
I	27	16 (59.3%)	0 (0%)	1 (3.7%)	2 (7.4%)	0 (0%)
II	681	292 (42.9%)	23 (3.4%)	28 (4.1%)	40 (5.9%)	11 (11.6%)
III	245	126 (51.4%)	15 (6.1%)	14 (5.8%)	13 (5.3%)	1 (0.4%)
IV	345	295 (85.5%)	38 (11.0%)	21 (6.1%)	34 (9.9%)	9 (2.6%)
Total	1298	729 (56.2%)	76 (5.9%)	64 (4.9%)	89 (6.9%)	21 (1.6%)
Therapy group						
TG-1	442	186 (42.1%)	10 (2.3%)	15 (3.4%)	27 (6.1%)	1 (0.2%)
TG-2	295	134 (45.4%)	12 (4.1%)	15 (5.1%)	14 (4.7%)	5 (1.7%)
TG-3	561	409 (72.9%)	54 (9.6%)	34 (6.1%)	48 (8.6%)	15 (2.7%)
Total	1298	729 (56.2%)	76 (5.9%)	64 (4.9%)	89 (6.9%)	21 (1.6%)

**Fig. 2:**
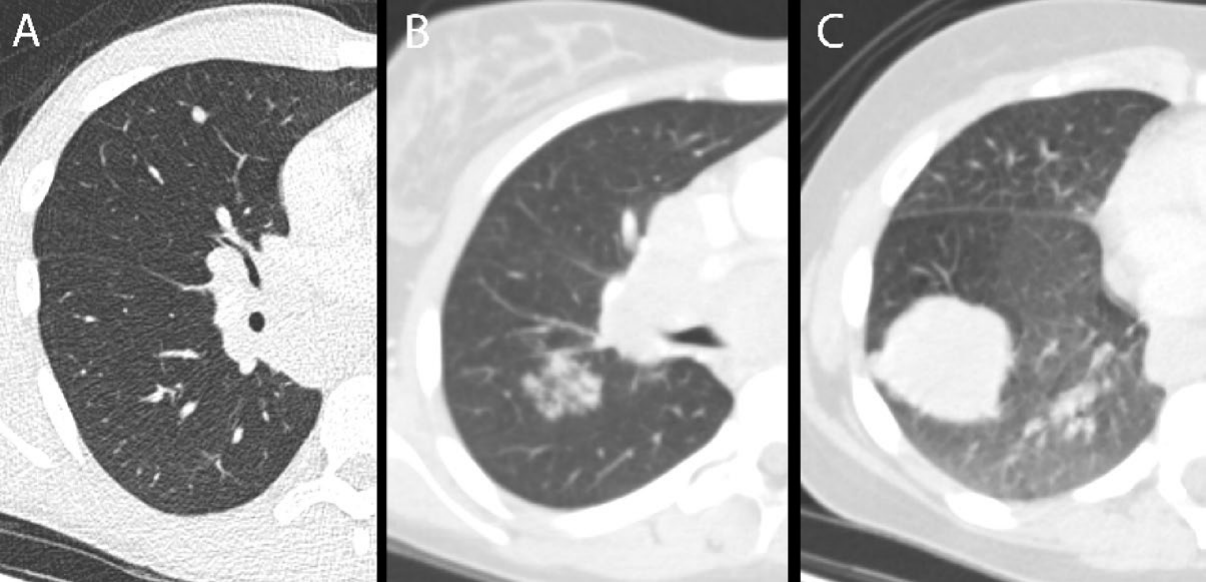
Different morphologic patterns of lung lesions in patients with pediatric classical Hodgkin lymphoma. Examples of different lesions present on initial staging CT images on axial slices at lung window settings (center: -600 HU; width: 1500 HU), that were classified according to the current recommended terms of the Fleischner Society. (**A**) nodules (**B**) consolidations (**C**) masses

Out of 3295 lesions analyzed, 2927 (89%) were nodules, making it the most common pattern of lung lesions. Only 38 (1%) masses, 117 (4%) consolidations, 83 (3%) ground-glass opacities, 104 (3%) parenchymal bands and 26 (< 1%) reticular opacities were identified (Fig. 2). The majority of lung lesions were found in patients with stage IV disease (Table 2).

Nodules and masses varied in diameter, ranging from 1 mm to 77 mm with a mean of 6.1 mm. The majority of 2456 (83.9%) nodules were smaller than 10 mm and 2744 (93.7%) nodules were smaller than 15 mm. Lesions showed a predominance in the right lung and an accentuation of caudal lung regions (right: 2045 (62%) [upper lobe: 605; middle lobe: 536, lower lobe: 905]; left: 1250 (38%) [upper lobe: 510; lower lobe: 739]).

Among the 782 patients with lung lesions, 251 (32%) had lesions confined to the right lung, 119 (15%) had lesions limited to the left lung, and 412 (53%) presented with bilateral lesions. Of all 3295 lesions, 1394 (42%) were subpleural and 910 (28%) centrilobular. Lesions located close to the hilum accounted for 314 (10%), while 677 (21%) lesions had pleural contact.

### III. Impact of lung lesions on PFS in TG-1 and TG-2

**Fig. 3:**
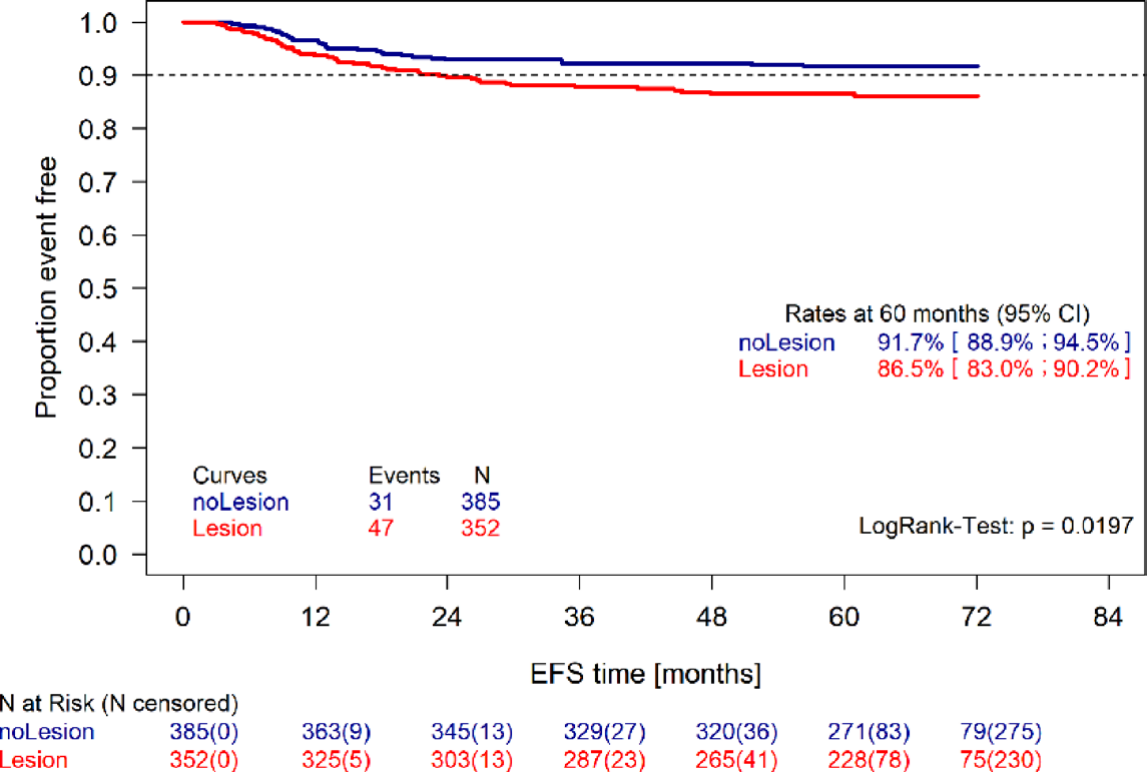
Comparison of progression-free survival between patients with and without lung lesions in the combined TG-1 and TG-2 cohort. Estimates of progression-free survival demonstrated lower 60-month PFS in patients with lung lesions (red) compared to those without lung lesions (blue). The target survival rate of 90% was not reached in the combined TG1 + 2 group with lung lesions; however, the corresponding confidence interval included the target value

In a combined analysis of patients in TG-1 and TG-2 (TG1 + 2; *n* = 737), PFS was significantly lower in patients with lung lesions (*n* = 352) compared to those without lesions (*n* = 385) from stages IA, IB, IIA, IAE, IBE, IIAE, IIB, or IIIA (85.5% [83.0–90.2%] vs. 91.7% [88.9–94.5%]; *p* = 0.0197) (Fig. 3). Notably, the group with lung lesions included only patients who were not considered to have lung involvement, as these patients would have been classified as stage IV and assigned to TG-3.

**Fig. 4:**
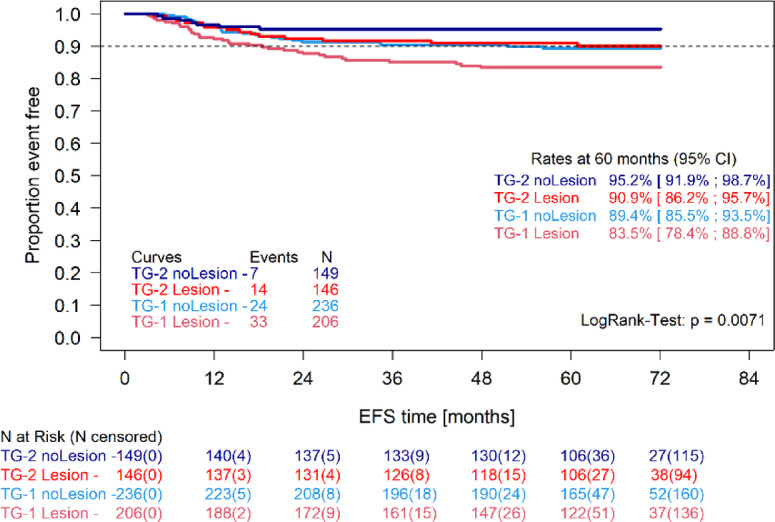
Comparison of progression-free survival in TG-1 and TG-2 stratified by the presence or absence of lung lesions. Estimates of progression-free survival showed a lower 60-month PFS in patients with lung lesions (TG-1: light red; TG-2: dark red) compared to patients without lung lesions (TG-1: light blue; TG-2: dark blue). The target rate of 90% was not reached in patients in TG-1 with lung lesions

This negative impact of lung lesions on PFS remained significant when TG-1 and TG-2 were analyzed separately (*p* = 0.0071). In TG-1, patients with lung lesions (*n* = 206) had a lower 60-month PFS compared to those without (*n* = 236) lesions (83.5% [78.4–88.8%] vs. 89.4% [85.5–93.5%]). Similarly, in TG-2, patients with lung lesions (*n* = 146) demonstrated lower PFS relative to patients without (*n* = 149) lesions (90.9% [86.2–95.7%] vs. 96.2% [91.9–98.7%]) (Fig. 4).

Given that nodules were the most frequent morphologic pattern, 60-month PFS was analyzed in the combined TG-1 + 2 cohort (*n* = 737) based on their presence. The group with nodules (*n* = 320; 43.4%) consisted of 186 patients from TG-1 and 143 from TG-2, presenting with nodules either alone or in combination with other patterns. The group without nodules (*n* = 417; 56.6%) served as the comparator, comprising patients with no lung lesions or exclusively non-nodular lesions. In this analysis, there was no statistically significant difference (*p* = 0.1417) in PFS between patients with (87.4% [83.7%; 91.2%]) and patients without (90.6% [87.8%; 93.5%]) nodules. The analysis of the impact of the number of nodules showed also no impact on PFS rates (*p* = 0.1149).

**Fig. 5:**
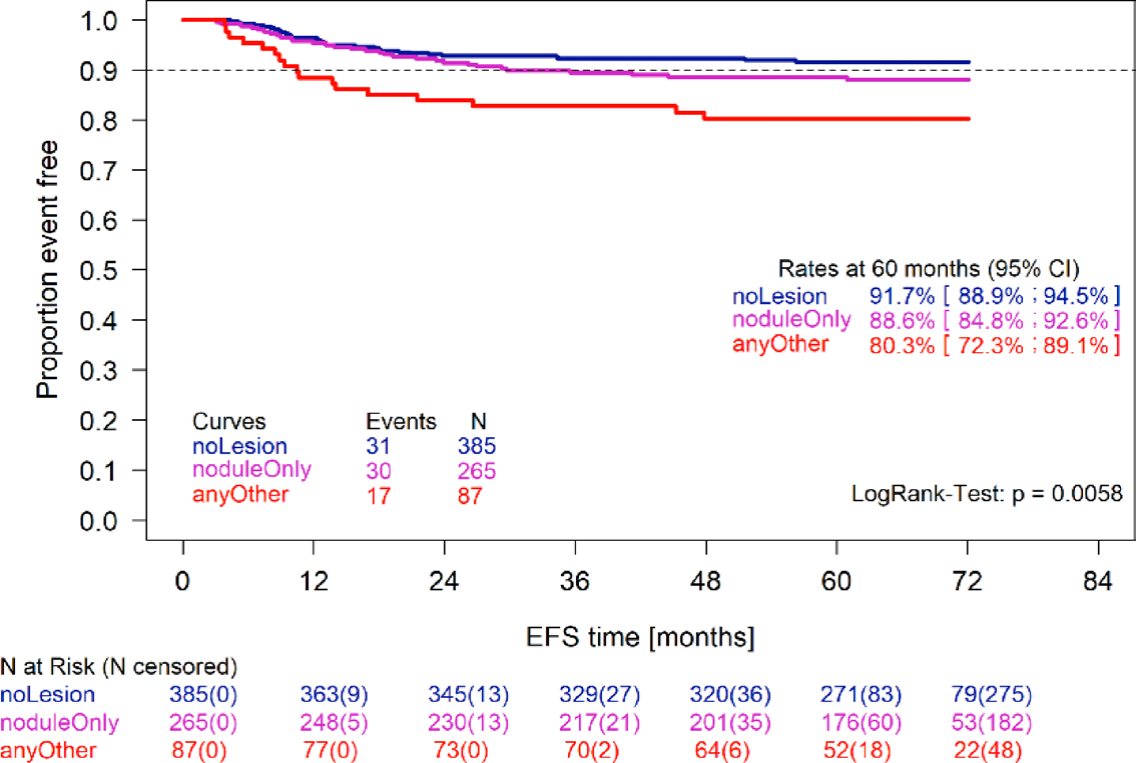
Comparison of progression-free survival in the combined TG-1 and TG-2 cohort stratified by the morphologic pattern of lung lesions. Estimates of progression-free survival of patients showed a significant difference among patients without lung lesions (blue), those presenting exclusively with nodules (pink), and those with at least one other lesion pattern (red). The latter group demonstrated a significantly lower 60-month PFS, which did not reach the 90% target rate

To assess the impact of different morphologic patterns on PFS in TG1 + 2, patients without lung lesions (*n* = 385) were compared to those presenting exclusively with nodules (*n* = 265) and those with at least one other lesion pattern (*n* = 87). PFS estimates demonstrated a significant difference between the groups (*p* = 0.0058), with 60-month PFS rates of 91.7% (88.9–94.5%) for patients without lesions, 88.6% (84.8–92.6%) for patients with nodules only, and 80.3% (72.3–89.1%) for patients with any other morphologic pattern (Fig. 5).

### IV. Impact of lung lesions on PFS in TG-3

The TG-3 cohort comprised 561 patients with advanced disease:, of whom 216 patients (38.5%) were stages IIBE, IIIAE, IIIB or IIIBE, and 345 (61.5%) patients were stage IV. Among the patients with TG-3 non-stage-IV disease, 58.8% (127/216) had lung lesions and in the TG-3 stage-IV cohort, 87.8% (303/347) patients had lung lesions (Fig. 1).

Among the 303 TG-3 stage IV patients with lung lesions, 59.1% (179/303) had lung lesions consistent with lung involvement, whereas 40.9% (124/303) had lung lesions that did not meet the criteria for lung involvement, but had at least one other organ involved. Of the 179 TG-3 stage IV patients with lung involvement, 32.4% (58/179) had at least one additional organ involved, while 67.6% (121/179) had lung involvement as the sole site of extranodal disease (Fig. 1).

**Fig. 6:**
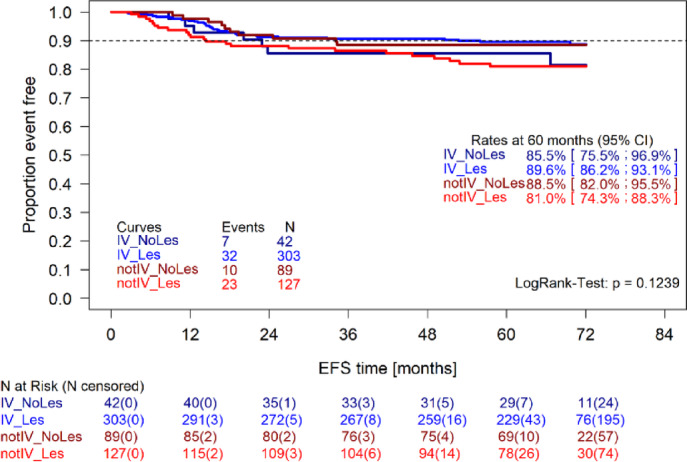
Comparison of progression-free survival of patients in TG-3 stratified by stage IV versus non-stage IV status and the presence of absence of lung lesions. Estimates of progression-free survival of patients with advanced disease demonstrated no significant difference among patients in TG-3 stage IV without lung lesions (dark blue), patients in TG-3 stage IV with lung lesions (light blue), patients in TG-3 non-stage IV without lung lesions (dark red), and TG-3 stage non-stage IV with lung lesions (light red). No statistically significant differences were observed between the four groups

To assess the impact of lung lesions on PFS within TG-3, four patient subgroups were compared: non-stage IV patients without lung lesions (*n* = 89), non-stage IV patients with lung lesions (*n* = 127), stage IV patients without lung lesions (*n* = 42), and stage IV patients with lung lesions (*n* = 303) with non-significant differences (*p* = 0.1239). In the non-stage IV cohort, patients with lung lesions showed a trend toward lower PFS compared to those without lesions (81.0% [74.3–88.3%] vs. 88.5% [82.0–95.5%]). In the stage IV cohort, patients without lung lesions (representing those with involvement of other extranodal sites such as liver or bone marrow) demonstrated slightly lower PFS than those with lung lesions (85.5% [75.5–96.9%] vs. 89.6% [86.2–93.1%]) (Fig. 6).

**Fig. 7:**
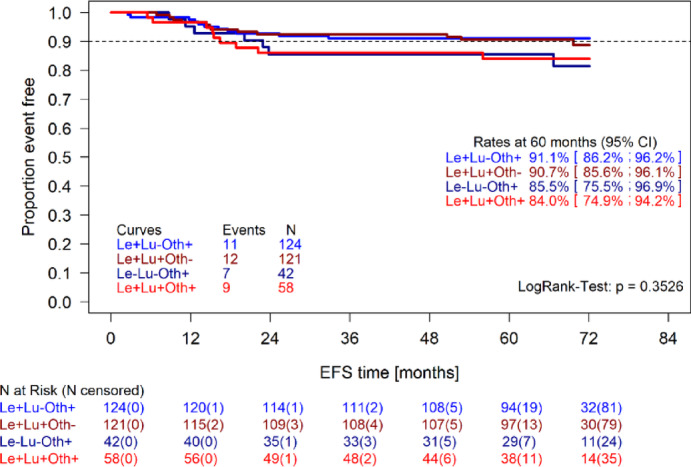
Comparison of progression-free survival in TG-3 stage IV patients according to the presence or absence of stage-IV-qualifying lung lesions and other organ involvement. Estimates of progression-free survival demonstrated no significant differences among patients in TG-3 stage IV with lung lesions not consistent with lung involvement but with at least one other organ involved (Le^+^Lu^−^Oth^+^; light blue), those with lung lesions consistent with lung involvement and no other organ involvement (Le^+^Lu^+^Oth^−^; brown), those without lung lesions but with at least one other organ involved (Le^−^Lu^−^Oth^+^; dark blue), and those with lung lesions consistent with lung involvement and at least one additional organ involved (Le^+^Lu^+^Oth^+^; red).

For a comprehensive evaluation of the EuroNet-PHL-C1 staging criteria for lung involvement and their impact on PFS, an additional subgroup analysis was performed within TG-3. Four stage IV groups were compared: patients with lung lesions not consistent with lung involvement but with at least one other organ involved (Le^+^Lu^−^Oth^+^; *n* = 124), patients with lung lesions consistent with lung involvement and no other organ involvement (Le^+^Lu^+^Oth^−^; *n* = 121), patients without lung lesions but with at least one other organ involved (Le^−^Lu^−^Oth^+^; *n* = 42), and patients with lung lesions consistent with lung involvement and at least one additional organ involved (Le^+^Lu^+^Oth^+^; *n* = 58). No significant difference in PFS was observed among the four groups (Le^+^Lu^−^Oth^+^: 91.1% [86.2–96.2%] vs. Le^+^Lu^+^Oth^−^: 90.7% [85.6–96.1%] vs. Le^−^Lu^−^Oth^+^: 85.5% [75.5–96.9%] vs. Le^+^Lu^+^Oth^+^: 84.0% [74.9–94.2%]; *p* = 0.3526) (Fig. 7).

## Discussion

Survivors of pediatric cHL are at high risk of adverse late-effects through therapy including secondary cancers, infertility and cardiovascular diseases. The current challenge is to maintain high cure rates while reducing radiation exposure and minimizing cumulative doses of chemotherapy to decrease adverse late-effects. The EuroNet­PHL­C1 trial demonstrated that RT can be omitted in patients with early-stage disease (TG-1) who respond adequately to OEPA induction, and in patients with intermediate or advanced-stage disease (TG-2 and TG-3) who respond adequately to OEPA induction when consolidated with COPP or COPDAC^7,8^.

The allocation of patients to TGs depends not only on the individual risk factors of high ESR, presence of bulky disease, B-symptoms and E-lesions, but also on the Ann Arbor stage determined by diagnostic imaging. Patients with extranodal involvement are assigned to Ann Arbor stage IV disease and subsequently to TG-3 in the EuroNet-PHL-C1 trial regardless of other prognostic factors. Therefore, clear staging definitions are critical to avoid upstaging and ensure consistency of staging. As the majority of participants in EuroNet-PHL-C1 (42.3%; 909/2102) were in TG-3 and 25.3% (531/2102) were stage IV, it is essential to analyze survival data by organ involvement to identify patients that may achieve the same high cure rates with the least amount of treatment possible and therefore refine current staging definitions. The staging definition used in EuroNet-pHL-C1 classified patients as stage IV as soon as the criteria for size and number of intrapulmonary lesions are met: the lung was considered involved if there were > 3 foci with a diameter of less than 10 mm within the whole lung or at least one intrapulmonary focus of a diameter ≥ 10 mm. Therefore, this definition did not take morphology or metabolic activity of lung lesions into account. Given the high prevalence of incidental lung lesions in children in general^27^, the challenge is to differentiate stage-IV-qualifying lung lesions from non-stage-IV-qualifying lung lesions, since it is not appropriate to equate having a lung lesion with having lung involvement, e.g. stage IV disease.

The present analysis of the prevalence, pattern and prognostic impact of lung lesions included 62% (1298/2012) of patients participating in the EuroNet-PHL-C1 trial. A comparison of baseline characteristics revealed no significant differences between included and non-included patients, except for the presence of bulky disease. Patients with bulky disease were significantly overrepresented in the analyzed cohort compared with those not included (40.6% vs. 32.3%) (*p* < 0.001). This overrepresentation of bulky disease may influence the generalizability of our findings, particularly for early-stage patients in whom bulky disease is less common. The higher proportion of bulky disease in the analyzed cohort is likely related to the fact that only patients who underwent German central imaging review - using different measurement criteria compared with the French central review - were included in this analysis. While this imbalance affects the composition of the cohort, the prognostic relevance of bulky disease itself is mitigated in contemporary, response-adapted treatment regimens, in which intensified chemotherapy or selective use of radiotherapy can compensate for increased tumor burden^41^. Therefore, although the distribution of bulky disease differs from the overall EuroNet-PHL-C1 population, a substantial impact on outcome interpretation is unlikely.

At initial presentation, lung lesions were present in 60.2% (782/1298) of patients in the analyzed cohort, highlighting their high prevalence. The most common patterns were nodules, followed by consolidations and ground-glass opacities. The impact of these lesions on PFS was analyzed separately for TG-1 and TG-2 from TG-3. This was necessary to perform a risk-adjusted analysis taking into account the intensity of therapy, as the impact of baseline characteristics on prognosis depends on the intensity of therapy in pHL^37,39^.

In the analysis of TG-1 and TG-2, patients without lung lesions were compared with those presenting with lung lesions that did not meet the EuroNet-PHL-C1 staging criteria for lung involvement^28^, as patients meeting these criteria would have been classified in TG-3. In both TG-1 and TG-2, patients with lung lesions had a slightly worse PFS compared to patients in the respective treatment group without lung lesions (Figs. 3 and 4). Notably, in TG-1, patients with lung lesions did not reach the predefined 5-year PFS target of 90% (83.5%), and unlike the combined TG1 + 2 cohort, the upper confidence limit (88.8%) remained below the 90% target. The confidence intervals for the other three groups in the separate analysis of TG-1 and TG-2 (TG-1 without lung lesions, TG-2 with lung lesions, and TG-2 without lung lesions) included the target rate. This observation is consistent with overall results from the EuroNet-PHL-C1 trial^7,8^: among TG-1 patients who responded adequately and were treated without radiotherapy (RT), the 5-year event-free survival (EFS) was 86.5% (95% CI: 83.3–89.8%), falling short of the predefined goal. In TG-1 patients with an inadequate early response who subsequently received RT, the 5-year EFS was 88.6% (95% CI: 84.8–92.5%). The omission of RT and limitation to only two cycles of chemotherapy likely contributed to the slightly lower PFS in TG-1, particularly in patients with subtle residual disease or higher baseline risk.

Regarding the impact of lung lesions in TG1 + 2, it is important to note that the presence of non-stage-IV-qualifying lung lesions at staging occurred in both TG-1 and TG-2 and did not influence treatment allocation in EuroNet-PHL-C1. Consequently, differences between TG-1 and TG-2 were driven more by other baseline characteristics and therapy intensity than by the presence of non-stage-IV-qualifying lung lesions. In TG-1, where patients with lung lesions did not reach the 90% PFS target, it is noteworthy that a substantial proportion of patients (37–60%) belonged to the higher-risk subgroup (defined by the presence of bulky disease > 200 mL or ESR > 30 mm). In the subsequent EuroNet-PHL-C2 trial, these patients would have been allocated to TL-2 and received two additional cycles of consolidation chemotherapy. ^7,8^ The presence of lung lesions in TG-1 and TG-2 may have contributed to minor differences in outcomes between groups; however, these findings do not alter the overarching conclusion that treatment intensity - rather than baseline lung lesions in patients with early- or intermediate-stage disease - was the primary determinant of PFS. A more detailed analysis, stratified by the morphologic pattern of lung lesions, revealed that the reduced PFS within TG1 + 2 was not observed in patients with intrapulmonary nodules alone. Instead, the lower PFS was confined to patients presenting with other lesion patterns, in whom the 5-year PFS target of 90% was not reached (Fig. 5). While non-nodule lesions were observed in the TG-1 and TG-2 cohorts in this retrospective analysis (Table 2), the imaging re-evaluation did not include access to detailed clinical information. Therefore, it was not possible to determine whether these findings had initially been attributed by treating physicians to infection, benign etiologies, or how those diagnostic decisions were made.

In the TG-3 cohort, lung lesions were highly prevalent (76.6%; 430/561), particularly in stage IV disease (87.8%; 303/345). Among stage IV patients with lung lesions, only 59.1% (179/303) patients met EuroNet-PHL-C1 criteria for lung involvement, while the remainder (40.9%; 124/303) represented non–stage-IV-qualifying lung lesions in patients who already had another extranodal site affected. Despite the high frequency of lung lesions, their presence did not significantly affect PFS in TG-3. Neither the comparison between stage IV and non–stage-IV patients with or without lung lesions (Fig. 6), nor the more granular analysis of stage IV subgroups distinguished by lung involvement and additional organ involvement (Fig. 7), revealed significant prognostic separation. Overall, these findings indicate that within advanced-stage disease with high treatment intensity, lung lesions - whether lung involvement-defining or not - do not independently influence PFS.

Comparison of the results of this analysis with previous studies is challenging because there is a paucity of literature on the prevalence and morphologic pattern of lung involvement in pHL in larger cohorts^22–24,42–46^. In 2004, Maturen et al. ^23^ firstly took account of the pediatric age group. They assessed the prevalence of pulmonary parenchymal lesions in children with HL, non-Hodgkin lymphoma and post-transplant lymphoproliferative disorders. They performed a 10-year retrospective analysis of 161 lymphoma patients including 82 (51%) patients with HL. Prevalence of pulmonary involvement was 12% (10/82). CT findings were described only for the inhomogeneous lymphoma collective. They found that pulmonary nodules were the most common lesions.

For pHL, studies about the impact of lung lesions on survival with respect to current treatment strategies are limited. An outcome analysis for participants in AHOD1331 (NCT02166463) was provided by Casey et al. who analyzed PFS and prognostic implications in 369 pediatric and adolescent young adults with stage IV disease, of whom 183 (50%) were treated with ABVE-PC (adriamycin, bleomycin, vincristine, etoposide, prednisone, cyclophosphamide) and 186 (50%) with Bv-AVE-PC (brentuximab vedotin, adriamycin, vincristine, etoposide, prednisone, cyclophosphamide). Isolated lung involvement was present in 185 patients (50%). They observed a nonsignificant difference in 3-year PFS of 88.0% (82.3%; 91.9%) for lung and 82.5% (75.8%; 87.5%) for multi-site involvement (*p* = 0.21). Bv-AVE-PC significantly improved PFS in stage IV patients compared to ABVE-PC (90.2% vs. 81.5%; *p* = 0.01). In patients with isolated lung involvement, there was a non-significant increase in PFS with Bv (91.2% vs. 84.7%; *p* = 0.16), while those with multi-site disease experienced significantly improved PFS with Bv (88.1% vs. 77.1%; *p* = 0.05)^47^. Across contemporary pHL treatment strategies, isolated lung involvement does not appear to confer a clear adverse prognostic impact. Current evidence suggests that lung lesions alone are not a strong negative prognostic marker, and outcomes are more strongly influenced by treatment regimen and overall disease burden than by lung involvement per se.

One possible explanation for the results of our analysis could be that some nodules are intrapulmonary lymph nodes simulating organ involvement and cannot be differentiated from true organ involvement by CT morphology^48,49^. Biopsy of lung lesions is not feasible for ethical reasons and because of its low diagnostic yield, leaving diagnostic uncertainty ^50^. Therefore, previous work on this topic suggested increasing the cut-off diameter of lung lesions to 10 mm without regards to the number of lesions^38^.

Limitations of the present study include its multicenter design and retrospective approach to re-review of images without a control group, so PFS analysis was only possible for the combination chemotherapy of OEPA and COPP or COPDAC. Since the treatment strategy limited RT to patients with inadequate PET-response at early response assessment after two cycles of OEPA, the effect of lung involvement and RT was not evaluated in the analysis of PFS. Nevertheless, restricting the analysis of lung lesions to morphological criteria seems sufficient, as 93.7% of the lesions at initial staging were smaller than 15 mm and thus unreliable for PET assessment of these lesions due to the limited detectability of PET, mainly caused by partial volume averaging and misregistration artifacts^33^. Furthermore, it needs to be taken into account that the EuroNet-PHL-C1 trial was conducted between 2009 and 2013 across 186 study centers, a period in which PET/CT technology and its standardization in pediatric oncology imaging were still evolving. Therefore, the analysis focused exclusively on the morphologic characteristics derived from CT scans, consistent with the EuroNet-PHL-C1 trial’s original staging principles for lung assessment. However, future trials should implement more uniform scanning protocols and a systematic analysis of PET data from lung lesions, leveraging the capabilities of modern PET/CT scanners. At present, the two largest pHL study groups, EuroNet and the Children’s Oncology Group (COG), define lung involvement based on the size and number of lung lesions, with COG additionally incorporating metabolic activity. Because there is no global consensus on staging criteria and current American and European definitions do not include morphologic patterns, expanding the criteria used within the global Staging Evaluation and Response Criteria Harmonization initiative for Childhood, Adolescent and Young Adult Hodgkin Lymphoma (SEARCH for CAYAHL)^28^.

While response assessment (e.g., changes in lesion size or metabolic activity after initial therapy) could also provide insights into the nature of lung lesions, the presented study focused on initial staging, which dictates upfront treatment decisions. Relying on response data to discern whether a lung lesion is pHL would postpone treatment decisions. While this approach might not interfere with the EuroNet-PHL-C1 treatment regimen, it might not be applicable to treatment regimens of other trials. Moreover, even with response data, definitive differentiation remains challenging: a decrease in size could indicate both response or resolution of an infection, while stability could signify a benign scar, a non-involved intrapulmonary lymph node or a non-responding lymphoma lesion. Furthermore, previous studies detected that even with newly occurring lung lesions during ongoing therapy in adequate responders are a quite frequent finding^38^. Therefore, for the critical purpose of initial staging and baseline risk stratification, this approach cannot be considered reliable.

As an additional limitation, not all participating European institutions provided images for central review in Germany, therefore the analysis presented covers only a subset of all patients enrolled in EuroNet-PHL-C1, which reduces the study population. Other limitations of the study were caused by the lack of a strict scanning protocol. This resulted in a limited image quality due to an oversized field of view and high slice thickness in a considerable number of cases.

In conclusion, the findings indicate that baseline lung lesions - particularly isolated intrapulmonary nodules - do not uniformly predict inferior outcomes in pHL and should not, on their own, be considered a strong adverse prognostic factor within contemporary treatment regimens. Instead, treatment intensity and overall disease burden remain the principal determinants of PFS. Nevertheless, the heterogeneity of lung findings and the observed disadvantage associated with non-nodule morphologies in early-stage and intermediate-stage disease underscore the need for more refined characterization of lung lesions although the EuroNet staging definition already captures the clinically relevant high-risk scenarios but may insufficiently differentiate between different morphologic patterns. Future trials should evaluate whether incorporating morphologic patterns into staging or risk stratification could improve prognostic precision. However, any modification of staging criteria would require prospective validation, ideally supported by harmonized imaging protocols and centralized PET/CT assessment. Until such evidence is available, the current size-/number-based definitions remain appropriate for clinical use, but future international efforts - such as within the global SEARCH for CAYAHL initiative - should explore refined criteria to enhance risk assignment while maintaining feasibility across treatment groups and imaging platforms.

## Data Availability

For original data please contact dietrich.stoevesandt@medizin.uni-halle.de.
